# NCI60 Cancer Cell Line Panel Data and RNAi Analysis Help Identify EAF2 as a Modulator of Simvastatin and Lovastatin Response in HCT-116 Cells

**DOI:** 10.1371/journal.pone.0018306

**Published:** 2011-04-04

**Authors:** Sevtap Savas, David O. Azorsa, Hamdi Jarjanazi, Irada Ibrahim-Zada, Irma M. Gonzales, Shilpi Arora, Meredith C. Henderson, Yun Hee Choi, Laurent Briollais, Hilmi Ozcelik, Sukru Tuzmen

**Affiliations:** 1 Fred A. Litwin Centre for Cancer Genetics, Samuel Lunenfeld Research Institute, Mount Sinai Hospital, Toronto, Canada; 2 Department of Pathology and Laboratory Medicine, Mount Sinai Hospital, Toronto, Canada; 3 Department of Laboratory Medicine and Pathobiology, University of Toronto, Toronto, Canada; 4 The Pharmaceutical Genomics Division, Translational Genomics Research Institute (TGen), Scottsdale, Arizona, United States of America; 5 The Clinical Translational Research Division, Translational Genomics Research Institute (TGen), Scottsdale, Arizona, United States of America; 6 Prosserman Centre for Health Research, Mount Sinai Hospital, Toronto, Canada; National Cancer Institute, United States of America

## Abstract

Simvastatin and lovastatin are statins traditionally used for lowering serum cholesterol levels. However, there exists evidence indicating their potential chemotherapeutic characteristics in cancer. In this study, we used bioinformatic analysis of publicly available data in order to systematically identify the genes involved in resistance to cytotoxic effects of these two drugs in the NCI60 cell line panel. We used the pharmacological data available for all the NCI60 cell lines to classify simvastatin or lovastatin resistant and sensitive cell lines, respectively. Next, we performed whole-genome single marker case-control association tests for the lovastatin and simvastatin resistant and sensitive cells using their publicly available Affymetrix 125K SNP genomic data. The results were then evaluated using RNAi methodology. After correction of the *p-*values for multiple testing using False Discovery Rate, our results identified three genes (*NRP1*, *COL13A1*, *MRPS31*) and six genes (*EAF2*, *ANK2*, *AKAP7*, *STEAP2*, *LPIN2*, *PARVB*) associated with resistance to simvastatin and lovastatin, respectively. Functional validation using RNAi confirmed that silencing of *EAF2* expression modulated the response of HCT-116 colon cancer cells to both statins. In summary, we have successfully utilized the publicly available data on the NCI60 cell lines to perform whole-genome association studies for simvastatin and lovastatin. Our results indicated genes involved in the cellular response to these statins and siRNA studies confirmed the role of the *EAF2* in response to these drugs in HCT-116 colon cancer cells.

## Introduction

Simvastatin and lovastatin are two statins traditionally used for lowering serum cholesterol levels. The statins are reversible inhibitors of the microsomal enzyme HMG-CoA reductase, which converts HMG-CoA to mevalonate. This is an early rate-limiting step in cholesterol biosynthesis. In humans, inhibition of HMG-CoA reductase by statins decreases intracellular cholesterol biosynthesis, which then leads to transcriptionally upregulated production of microsomal HMG-CoA reductase and cell surface LDL receptors. However, simvastatin and lovastatin differ in some important aspects concerning the degree of metabolism and the number of active and inactive metabolites [1]. More recently, statins have gained significant notice as anticancer agents based on preclinical evidence of their antiproliferative, proapoptotic, anti-invasive and radiosensitizing properties [2], [3], [4], [5], [6]. The role of statins in cholesterol metabolism can explain their potential cytotoxic characteristics.

Cholesterol is a key lipid that accumulates in membrane micro-domains called lipid rafts. Lipid rafts play an important role in signal transduction that triggers cell growth, survival and many other processes that are correlated with cancer. Cholesterol accumulation in tumors has been demonstrated by a number of studies in the past [7], [8], [9], [10]. Accumulation of cholesterol within lipid raft micro-domains of the plasma membrane may play a role in stimulating signal transduction pathways. Freeman and Solomon (2004) have proposed that increase in cholesterol in prostate tumor cell membrane, which may result from an increase in circulating levels or from deregulation of endogenous synthesis, give rise to the coalescence of the raft domains [7]. This in turn could have an effect on the segregation of positive regulators of oncogenic signaling within rafts, while keeping negative regulators in the fluid mosaic membrane fraction [7]. It was further proposed that the study of the function of lipid rafts in prostate cancer cells might provide insight into the role of circulating cholesterol in malignant growth and on the potential relationship between diet and aggressive disease. Therefore, characterization of proteins within cholesterol-rich micro domains may serve to better clarify the signaling pathways, which will lead to the identification of novel biomarkers for disease progression and new targets for cancer therapy.

Variable response to drug treatment, such as resistance, is a serious health concern. Several factors, such as age and diet, are implicated in chemotherapeutic resistance by influencing the drug adsorption, transportation, metabolism, and their physiological actions. Genetic factors are also involved in drug resistance. For example, genetic variations that cause alterations in gene function and expression are implicated in drug resistance [11], [12]. Therefore, for an optimal treatment efficacy, we need to know the genes associated with drug resistance as well as their profiles in each patient (personalized medicine). In this regard, the NCI60 cell line panel forms a promising tool to discover new cancer drugs. The NCI60 cell line panel is established from a variety of tumors in order to identify the compounds that can kill cancer cells [13]. Thus far, this cell line panel has been exposed to over 100,000 different compounds and the cellular responses in the form of growth rates have been measured. Using NCI60 cell lines, L-Asparaginase was identified as effective in killing a subset of ovarian carcinomas [14]. This panel was also used in the development of bortezomib for treatment of myeloma [13].

The experimental results obtained on the NCI60 cell lines are compiled at the Developmental Therapeutics Program (DTP) website [13]. In addition to pharmacological data mentioned above, other data for NCI60 cell lines is available at the DTP website, such as the genotypes of the Affymetrix 125K chip single nucleotide polymorphisms (SNPs). Affymetrix 125K SNP chip platform has a dense set of SNPs (∼124,000) and is utilized to identify the genomic regions that are associated with disease predisposition and variable treatment response. Previously, we have used the NCI60 cell line data to investigate drug resistance genes in human genome [15],[16],[17]. In this study, we took advantage of both the available pharmacological and genomic data on the NCI60 cell lines to identify the genes associated with cytotoxic resistance to simvastatin and lovastatin and performed functional studies using siRNA to validate *EAF2* as a modulator of statin activity.

## Materials and Methods

### Lovastatin and simvastatin Resistant and Sensitive NCI60 Cell Lines

**Figure 1:**
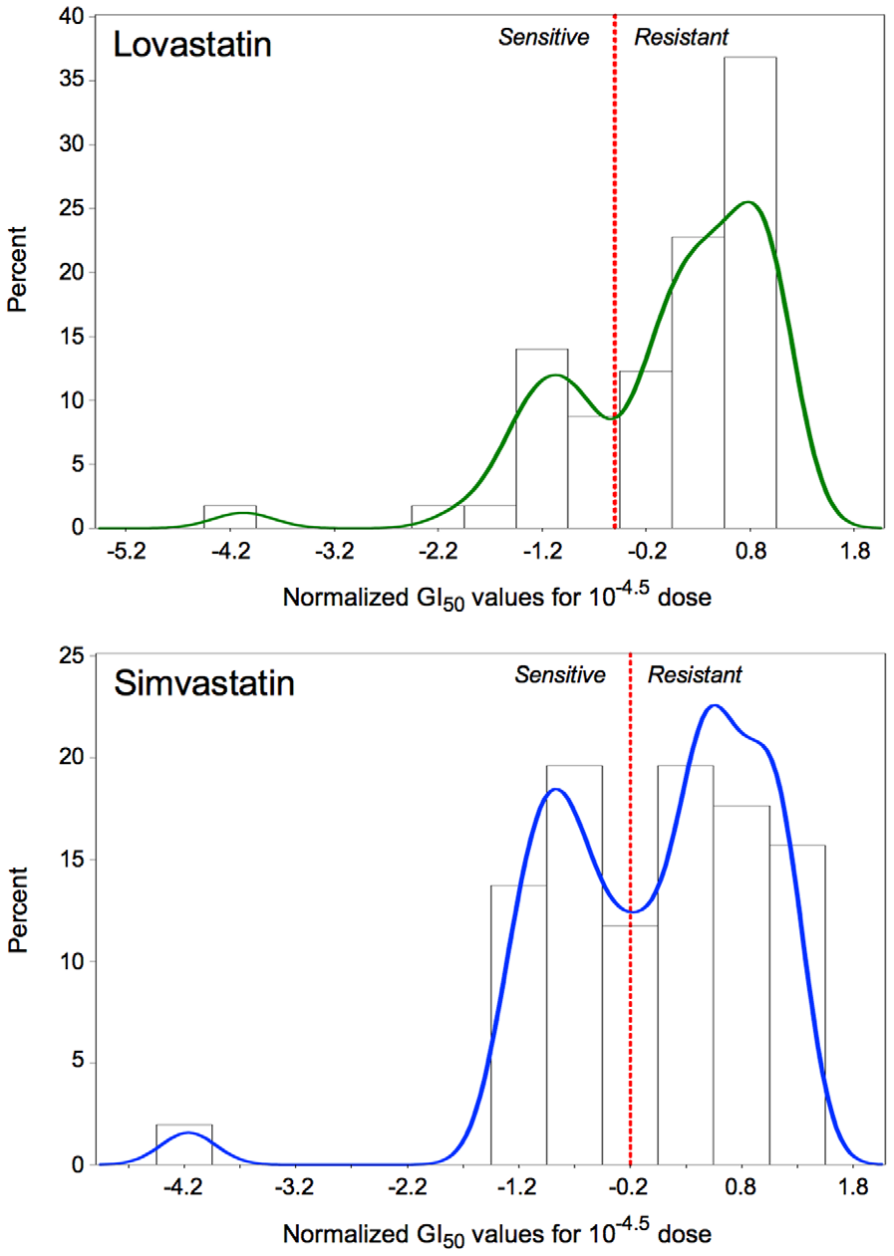
Distribution of NCI60 cell lines with respect to their response to treatment with lovastatin or simvastatin. The density function showed two major modes for each drug. The visual antimodes were used as a cut-off value at −0.2 for simvastatin and −0.3 for lovastatin to define sensitive and resistant NCI60 cell lines.

We have followed a previously developed approach to perform the whole-genome case-control association study [15], [16], [17]. Briefly, we have utilized the publicly available data on the NCI60 cell line panel posted at the Developmental Therapeutics Program (DTP) website of NCI/NIH (http://dtp.nci.nih.gov/index.html). First, we downloaded the GI_50_ data (the amount of the drugs required to inhibit growth by 50%) at the 10^−4.5^ M dose for the cell lines. Next, we categorized the cells as relatively resistant or sensitive after normalizing the log_10_ of GI_50_ to obtain a mean of zero and standard deviation of one as previously described [15], [16], [17]. The standardized GI_50_ values were then analyzed by the SAS 9.1 (PROC UNIVARIATE) with a non-parametric distribution test (density kernel estimation) with estimated bandwidths of 0.2476 and 0.2896 as well as asymptotic mean integrated squared errors (AMISE) of 0.0224 and 0.0172, for simvastatin and lovastatin, respectively. The visual antimodes were used as a cut-off value at −0.2 for simvastatin and −0.3 for lovastatin to define sensitive (controls) and resistant (cases) NCI60 cells (**Figure 1**). In the case of simvastatin, there were 19 sensitive and 32 resistant cell lines in the panel (**Table S1**). There were 16 sensitive and 41 resistant cell lines in the NCI60 panel for lovastatin (**Table S2**).

### Whole-Genome Case-Control Association Study

We downloaded the Affymetrix 125K SNP data (that had approximately 124,000 SNPs spaced with a median intermarker distance of 8.5 kilobases) from the DTP website (http://dtp.nci.nih.gov/mtargets/download.html) [18]. Whole-genome single marker case-control association tests for the lovastatin and simvastatin resistant and sensitive cells was performed by the PLINK software [19] using the standard chi-square test. Only the SNPs that have been genotyped in at least 75% of the cells and had a minimum minor allele frequency of 2% (n = 79,622) were included in this study and were used for the association testing. In order to decrease the chance for false-positive associations, a correction for multiple testing, *i.e*. the False Discovery Rate (FDR) proposed by Benjamini and Hochberg (FDR_BH) [20] was also performed by the PLINK software. Results with *p* values <0.05 were considered significant.

Information related to genomic locations (genic *versus* intergenic) of SNPs were either retrieved from the dbSNP database [21] or by blasting the SNP-flanking sequences against the human genome and by visualizing using the NCBI Map Viewer option [22].

### Epistasis (SNP-SNP Interaction) Analysis

Logistic regression models were used to analyze two-way interactions among the SNPs associated individually with simvastatin or lovastatin resistance, assuming an additive model for each SNP. For testing SNP-SNP interactions, we used the likelihood ratio test approach by comparing the fit of two models, one with the SNP main effects only and the other with the main effects and two-way interaction effect. The corresponding p-values were adjusted for multiple testing by the FDR_BH [20] method.

### Cell Culture

The human colon cancer cell lines HCT-116 and HT-29 were obtained from the American Type Culture Collection (Manassas, VA). HCT-116 is a tumorigenic colorectal carcinoma cell line established from a primary tumor [23]. HT-29 is a colon adenocarcinoma grade II cell line established from primary tumor cells [24]. Cells were grown in Dulbecco's modified Eagle medium (DMEM) supplemented with 10% FBS, 2 mM L-glutamine, 100 IU/ml penicillin G, and 100 µg/ml streptomycin. All media reagents were obtained from Invitrogen (Carlsbad, CA). The cell lines were routinely maintained at 37°C in a humidified 5% CO_2_ atmosphere.

### Functional validation for lovastatin and simvastatin sensitization

For siRNA and drug studies, cells were transfected with siRNA by reverse transfection in 384-well plates as previously described [25]. Briefly, siRNA was printed onto 384-well plates in 2 µl volume. Diluted siLentFect reagent (BioRad, Hercules, CA) in OptiMEM (Invitrogen) was added to the wells and allowed to complex with siRNA for 30 min at room temperature. HCT-116 or HT-29 cells were resuspended in growth media without antibiotics and added to plates at a final concentration of 1000 cells/well. Plates were incubated at 37°C with 5% CO_2_. After 24 hours, varying concentrations ranging from 12 nM to 667 µM of either lovastatin or simvastatin were added to the assay plates and incubated for an additional 72 hours. Total viable cell number was determined by the addition of Cell Titer Glo (Promega, Madison, WI) and relative luminescence units (RLU) were measured using an EnVision plate reader (Perkin-Elmer, Wellesley, MA). Drug effect was calculated by dividing the average of the RLU values for the drug treated wells by the average of the RLU values for vehicle treated wells. The GI_50_ values were determined using GraphPad Prism (GraphPad Software, San Diego, CA) and values were shown as calculated GI_50_+/−95% confidence interval. Statistical analysis of the data was done using two-tailed paired Student's *t* test. *P*<0.05 was considered significant.

### Validation of Gene silencing by Quantitative Real-time PCR (qPCR)

Total RNA from the cell lines was isolated using Qiagen's RNEasy Kit from Qiagen Inc. (Valencia, CA). RNA concentration was determined using NanoDrop-1000 according to the manufacturer's recommendations. iScript cDNA Synthesis Kit from Bio-Rad Inc. (Hercules, CA) was used to prepare cDNA from 500 ng of each sample. Relative mRNA expression was measured using TaqMan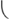 Gene Expression Assays from ABI Inc. (Foster City, CA) under manufacturer's recommended conditions on the Opticon 2 PCR System (Bio-Rad Inc., Hercules, CA). Relative quantification of gene expression was accomplished in triplicate. qPCR reactions were prepared in 96-well formatted qPCR plates from Bio-Rad (Hercules, CA). The reactions were prepared in singleplexes, triplicates of each sample with triplicates of endogenous controls, and non-template controls (NTC). Normalization was done in the presence of a reference gene, GAPDH, and the relative quantification of the gene expression changes were analyzed using the ΔΔCt method [26], [27].

## Results

### Genome wide associations studies of the NCI60 panel for lovastatin and simvastatin response

Using the NCI60 cancer-screening data for simvastatin and lovastatin, cell lines were categorized as relatively sensitive or resistant (**Figure 1**). The results obtained from the whole-genome case-control association studies for simvastatin and lovastatin are summarized in Tables 1 and 2, respectively. Eight SNPs were associated with resistance to simvastatin. These SNPs were located on chromosomes 8q, 9p (two SNPs), 10p, 10q, 13q (two SNPs) and 14p. Five of the SNPs were located in intergenic regions, whereas the remaining three SNPs were located in introns of known genes (Table 3), namely, intron 6 of *NRP1* (neurophilin), intron 37 of *COL13A1* (type XIII collagen, alpha 1), and intron 6 of *MRPS31* (mitochondrial ribosomal protein S31). Two intergenic SNPs, rs4129864 and rs1343844 were located very close (7387 base pairs away from each other), suggesting they were likely to be linked with each other.

**Table 1:** SNPs and genes associated with resistance to simvastatin.

SNP ID (Affy.)	dbSNP ID (rs number)	Chr. location	Alleles	Minor allele in NCI60 panel (number of alleles*)	Minor allele frequency in resistant cell lines (number of alleles*)	Minor allele frequency in sensitive cell lines (number of alleles*)	CHISQ	Unadjusted p value	Odds ratio	Lower confidence interval (95)	Upper confidence interval (95)	FDR_BH	Genomic location
2806028	rs6990201	8q	G/A	G (12/72)	0 (0/44)	0.4286 (12/28)	22.63	1.97E-06	n/a	n/a	n/a	0.02002	between LOC728724 and TMEM75
2846981	rs4129864	9p	C/G	C (13/76)	0 (0/46)	0.4333 (13/30)	24.05	9.40E-07	n/a	n/a	n/a	0.01437	between SH3GL2 and ADAMTSL1
2846980	rs1343844	9p	T/A	T (14/72)	0.04167 (2/48)	0.5 (12/24)	21.46	3.62E-06	0.04348	0.008551	0.2211	0.02763	between SH3GL2 and ADAMTSL1
**308318**	**rs1888690**	**10p12**	**G/C**	**G (13/56)**	**0 (0/36)**	**0.65 (13/20)**	**30.47**	**3.38E-08**	n/a	n/a	**n/a**	**0.001034**	**intron 6 of NRP1**
**348114**	**rs2394615**	**10q22**	**A/G**	**A (18/60)**	**0.08333 (3/36)**	**0.625 (15/24)**	**20.12**	**7.28E-06**	**0.05455**	**0.0129**	**0.2307**	**0.04447**	**intron 37 of COL13A1**
713311	rs1989252	13q14	T/C	T (23/74)	0.09091 (4/44)	0.6333 (19/30)	24.5	7.43E-07	0.05789	0.01629	0.2057	0.01353	intron 2 of LOC283507 (SUGT1L1), a pseudogene
**713232**	**rs7322754**	**13q14**	**A/C**	**A (20/68)**	**0.09524 (4/42)**	**0.6154 (16/26)**	**20.93**	**4.77E-06**	**0.06579**	**0.01796**	**0.241**	**0.02897**	**intron 6 of MRPS31**
895969	rs2025074	14p	T/C	T (13/66)	0.02381 (1/42)	0.5 (12/24)	21.9	2.88E-06	0.02439	0.002872	0.2071	0.02623	between SLC24A4 and CPSF2

Genome wide association test results for Simvastatin. n/a: not applicable, as the minor allele frequency of the alleles in one group (the resistant cell line group) is 0, these calculations cannot be performed.

*Please note that the number of alleles may differ in each marker due to missing genotypes in the panel.

**Table 2:** SNPs and genes associated with resistance to lovastatin.

SNP ID (Affy.)	dbSNP ID (rs number)	Chr. location	Alleles	Minor allele in NCI60 panel (number of alleles*)	Minor allele frequency in resistant cell lines (number of alleles*)	Minor allele frequency in sensitive cell lines (number of alleles*)	CHISQ	Unadjusted p value	Odds ratio	Lower confidence interval (95)	Upper confidence interval (95)	FDR_BH	Genomic location
**1840275**	**rs2332056, rs4339143**	**3q13**	**T/C**	**T (11/74)**	**0.01852 (1/54)**	**0.5 (10/20)**	**26.74**	**2.33E-07**	**0.01887**	**0.002168**	**0.1642**	**0.00712**	**intron 5 of EAF2**
**2032591**	**rs7667099**	**4q25**	**C/T**	**C (28/86)**	**0.1774 (11/62)**	**0.7083 (17/24)**	**22.21**	**2.44E-06**	**0.08881**	**0.02971**	**0.2655**	**0.02485**	**intron 2 of ANK2**
2123836	n/a	5p	G/A	G (8/88)	0 (0/64)	0.3333 (8/24)	23.47	1.27E-06	n/a	n/a	n/a	0.01939	between LOC340094 and LOC729099
**2458853**	**rs3777486**	**6q23**	**T/C**	**T (5/78)**	**0 (0/62)**	**0.3125 (5/16)**	**20.7**	**5.37E-06**	n/a	n/a	**n/a**	**0.04111**	**intron 1 of AKAP7**
2379872	n/a	6p	A/G	A (18/72)	0.1 (5/50)	0.5909 (13/22)	19.64	9.37E-06	0.07692	0.02192	0.2699	0.0425	intron 1 of C6orf142
2415786	n/a	6q	G/A	G (11/82)	0.03333 (2/60)	0.4091 (9/22)	19.57	9.71E-06	0.04981	0.009604	0.2583	0.0425	between CNR1 and LOC644119
**2601462**	**n/a**	**7q**	**T/C**	**T (6/72)**	**0 (0/54)**	**0.3333 (6/18)**	**19.64**	**9.37E-06**	n/a	n/a	**n/a**	**0.0425**	**intron 2 of STEAP2**
361943	rs4298845	10q	C/G	C (13/76)	0.05357 (3/56)	0.5 (10/20)	20.71	5.33E-06	0.0566	0.01319	0.2429	0.04111	between NRG3 and LOC728027
678678	rs997269	12q	G/A	G (21/82)	0.1167 (7/60)	0.6364 (14/22)	22.82	1.78E-06	0.07547	0.02335	0.2439	0.03407	between TMEM132C and LOC644489
675821	rs4765299	12q	T/C	T (10/80)	0.01724 (1/58)	0.4091 (9/22)	22.39	2.22E-06	0.02534	0.002946	0.218	0.03407	between LOC644152 and LOC728171
749272	rs768826	13q	T/C	T (25/70)	0.2 (10/50)	0.75 (15/20)	18.82	1.44E-05	0.08333	0.02444	0.2841	0.03275	between DACH1 and LOC647277
733322	rs831223	13q	C/T	C (6/90)	0 (0/66)	0.25 (6/24)	17.68	2.62E-05	n/a	n/a	n/a	0.03673	between DIAPH3 and LOC341689
726318	n/a	13q	T/C	T (6/88)	0 (0/64)	0.25 (6/24)	17.17	3.42E-05	n/a	n/a	n/a	0.04159	between OLFM4 and LOC387930
888232	rs2888473	14q	T/A	T (12/74)	0.05357 (3/56)	0.5 (9/18)	19.98	7.82E-06	0.0566	0.01281	0.25	0.02856	between FLRT2 and LOC283584
880180	n/a	14q	G/A	G (16/78)	0.08621 (5/58)	0.55 (11/20)	19.62	9.45E-06	0.07719	0.02164	0.2753	0.02876	between LOC730007 and DIO2
814584	rs1950874	14p	A/G	A (12/84)	0.03333 (2/60)	0.4167 (10/24)	20.57	5.74E-06	0.04828	0.009492	0.2455	0.02856	between NOVA1 and OR7K1P
819408	rs1033706	14p	C/T	C (17/82)	0.08621 (5/58)	0.5 (12/24)	17.69	2.60E-05	0.09434	0.02794	0.3186	0.03673	between PRKD1 and SYF2P (pseudogene)
1621189	n/a	21q	G/A	G (8/88)	0 (0/64)	0.3333 (8/24)	23.47	1.27E-06	n/a	n/a	n/a	0.02321	between CYCSP42 and USP25
1009300	rs1019553	16p	G/T	G (4/74)	0 (0/60)	0.2857 (4/14)	18.12	2.07E-05	n/a	n/a	n/a	0.03673	between PRM1 and C16orf75
1164490	rs7233911	18p	C/T	C (17/80)	0.1 (6/60)	0.55 (11/20)	18.15	2.04E-05	0.09091	0.02685	0.3078	0.03673	between LOC284230 and ZNF519
**1152242**	**rs641287**	**18p11**	**A/T**	**A (17/84)**	**0.1061 (7/66)**	**0.5556 (10/18)**	**17.7**	**2.58E-05**	**0.09492**	**0.02814**	**0.3202**	**0.03673**	**intron 4 of LPIN2**
1280595	rs1650937	19q	A/C	A (9/80)	0.01724 (1/58)	0.3636 (8/22)	19.17	1.20E-05	0.0307	0.003542	0.2661	0.03122	located in LOC204800 (a pseudogene)
1621191	rs2823460	21q	A/G	A (7/78)	0 (0/54)	0.2917 (7/24)	20.28	6.69E-06	n/a	n/a	n/a	0.02856	between USP25 and CYCSP42
1625348	rs2824643	21q	T/C	T (9/82)	0.01724 (1/58)	0.3333 (8/24)	17.36	3.10E-05	0.03509	0.004081	0.3017	0.04036	between LOC643081 and CHODL
**1709633**	**n/a**	**22q**	**A/G**	**A (27/88)**	**0.1719 (11/64)**	**0.6667 (16/24)**	**20.09**	**7.38E-06**	**0.1038**	**0.03564**	**0.3021**	**0.02856**	**intron 11 of PARVB**

Genome wide association test results for Lovastatin. n/a: not applicable, as the minor allele frequency of the alleles in one group (the resistant cell line group) is 0, these calculations cannot be performed.

*Please note that the number of alleles may differ in each marker due to missing genotypes in the panel.

**Table 3:** Summary of the biological processes, molecular functions, and cellular location of the proteins corresponding to the genes found associated with simvastatin and lovastatin resistance in this study.

Drug	Gene (Entrez Gene ID)	GO Biological Processes	GO Molecular Function	GO Cellular Component
**Simvastatin**	NRP1 (8829)	Axon guidance, cell-cell signaling, organ morphogenesis, positive regulation of cell proliferation, signal transduction	Protein binding, vascular endothelial growth factor receptor activity	Membrane fraction
	COL13A1 (1305)	Cell-cell adhesion, cell-matrix adhesion, endochondral ossification	Extracellular matrix structural constituent, heparin binding, protein binding	Collagen XIII, Plasma membrane
	MRPS31 (10240)	-	-	Mitochondrion
**Lovastatin**	EAF2 (55840)	-	Protein binding	-
	ANK2 (287)	-	Protein binding, structural constituent of cytoskeleton	Actin cytoskeleton, membrane
	AKAP7 (9465)	Intracellular signaling cascade, ion transport, protein localization	Protein kinase A binding	Apical plasma membrane, lateral plasma membrane, plasma membrane
	STEAP2 (261729)	Endocytosis, Golgi to plasma membrane transport, regulated secretory pathway, response to hormone response	Transporter activity	Cytosol, early endosome, integral to Golgi membrane, plasma membrane, trans-golgi network transport vesicle, vesicular fraction
	LPIN2 (9663)	no entry	no entry	no entry
	PARVB (29780)	-	Protein binding	-

Gene IDs are obtained from the Entrez Gene resource.

(http://www.ncbi.nlm.nih.gov/sites/entrez?db=gene) of the NCBI. The biological processes, molecular functions, and cellular component information is obtained from the Gene Ontology (GO) database [44].

In the case of lovastatin, a total of 25 SNPs were associated with its resistance. Six of these SNPs were located in known or predicted genes (Tables 2 and 3): in intron 5 of *EAF2* (ELL associated factor 2), in intron 2 of *ANK2* (neural ankyrin 2), in intron 1 of *AKAP7* (protein kinase A anchor protein 7), in intron 4 of *LPIN2* (Lipin 2), in intron 2 of *STEAP2* (six transmembrane epithelial antigen of the prostate 2), and in intron 11 of *PARVB* (parvin beta).

We further studied a possible SNP-SNP interaction for the simvastatin and lovastatin resistance SNP sets using logistic regression analysis and did not detect any statistically significant genetic interaction assuming additive genetic model after the FDR_BH adjustments. However, considering the small sample size, it should also be noted that our study did not have sufficient power, thus, these results should be interpreted cautiously. Pathway analysis did not identify direct interactions between the protein products of the genes in the simvastatin or lovastatin lists.

### Functional studies identifies EAF2 as a modulator of lovastatin and simvastatin

**Figure 2:**
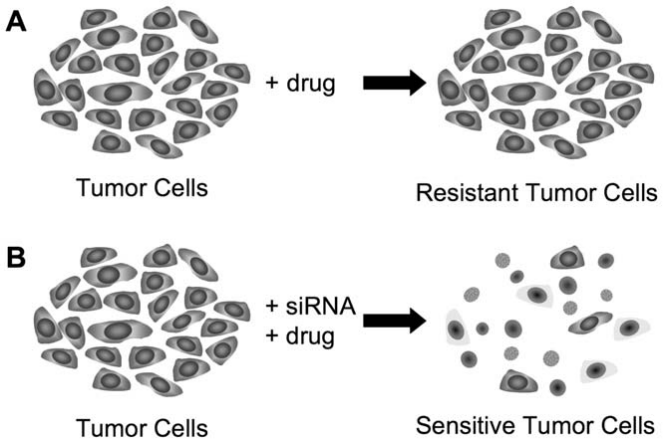
Schematic representation of reversal of drug resistance upon gene silencing by siRNAs. No biological relationship between the drug resistance and the genes/SNPs identified in the GWAS study was previously identified. Therefore, herein we hypothesize that if the functions of these genes are required for the resistance to these drugs, then, knocking-down their gene expression using siRNAs will disrupt the function of the genes, which will reverse the resistance and make the cells sensitive to these drugs again. (A) The tumor cell line is resistant upon exposure to drug. (B) Upon treatment with siRNA to candidate gene targets in addition to drug, we predict that the tumor cell line would become sensitized to the drug leading to increased cell death. This model is based on the hypothesis that the specific gene function is required for the drug resistance.

In order to test the validity of the positive GWAS results, we performed functional studies. We focused on the SNPs located in (intronic regions of) genes involved in simvastatin (3 SNPs) and lovastatin (6 SNPs) resistance (Tables 1 and 2). An extensive literature search did not reveal known functional consequences of these SNPs on gene expression or protein function. Thus, the direct biological relationships between these SNPs and resistance to simvastatin and lovastatin remained unknown. However, since our GWAS results have indicated an association of these genes with resistance to simvastatin or lovastatin, we hypothesized that the functions of these genes were somehow associated with drug resistance. Under this hypothesis, down regulation of the gene expression reverses the observed resistance and makes these cells sensitive to these drugs again, resulting in increased cellular toxicity and death (**Figure 2**). Therefore, we performed drug response and gene silencing using RNAi methodology studies on two colon cancer cell lines HCT-116 and HT-29, which were selected since they were included in the NCI60 set as well as for their good transfection efficiency and their response to the two statins. Based on the DTP drug response data, our analysis classified HCT-116 as relatively resistant to simvastatin (**Table S1**); however, lovastatin data was not available for this cell line. On the other hand, HT-29 was found to be relatively resistant to both simvastatin and lovastatin (**Tables S1 and S2**). Initially, both cell lines were treated with siRNA targeting genes identified in our analysis followed by treatment with two low doses of either simvastatin or lovastatin, which indicated four of the genes, *MRPS31*, *COL13A*, *EAF2*, *AKAP7* as potential modulators of drug response (Data not shown).

**Figure 3:**
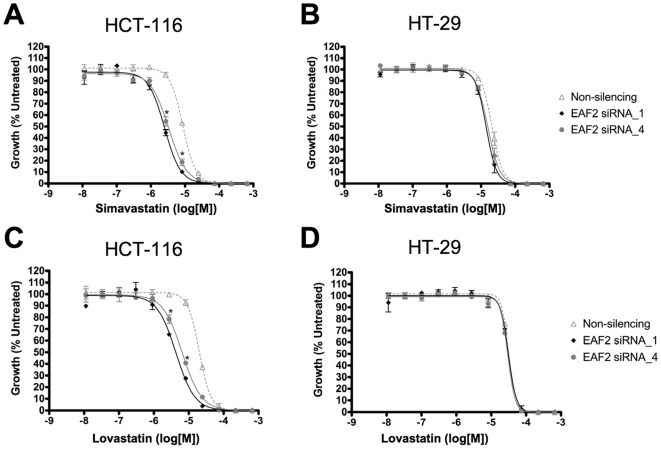
Effect of *EAF2* silencing on response to lovastatin and simvastatin. HCT-116 cells (A & C) and HT-29 cells (B & D) were transfected with siRNA targeting *EAF2* by reverse transfection. At 24 hours, the cells were treated with varying doses of either simvastatin (A & B) or lovastatin (C & D) ranging from 12 nM to 667 µM. Cell number was determined at 72 hours of drug exposure using Cell Titer Glo. Silencing of *EAF2* with specific siRNA significantly affected the response of HCT-116 cells compared to control non-silencing siRNA (*p<0.0002* for both EAF2_1 and EAF2_4 siRNAs shown by *) at doses 2.7 µM and 8.2 µM.

Further drug dose response studies were done using siRNA duplexes targeting *MRPS31* and *COL13A*, which were associated with resistance to simvastatin, and *EAF2* and *AKAP7*, which were associated with resistance to lovastatin. These genes were silenced by siRNA and treated with varying doses of either simvastatin or lovastatin ranging from 12 nM to 667 µM. Silencing of *MRPS31*, *AKAP7* and *COL13A* did not affect the response to either drug under the experimental conditions applied (data not shown), while silencing of *EAF2* significantly reduced the GI_50_ of simvastatin and lovastatin-treated HCT-116 cells, and thus reduced the resistance of this cell line to these drugs (**Figure 3**). For HCT-116 cells treated with simvastatin and siRNA, the GI_50_ (with 95% confidence limits) shifted from 8.6+/−0.3 µM for non-silencing siRNA (negative control) to 2.5+/−0.2 µM and 3.3+/−0.3 µM for EAF2_1 and EAF2_4 siRNA, respectively. Similarly, for HCT-116 cells treated with lovastatin the GI_50_ shifted from 20.1+/−0.9 µM for non-silencing siRNA to 4.3+/−0.5 µM and 6.5+/−0.5 µM for EAF2_1 and EAF2_4 siRNA, respectively. Furthermore, efficient siRNA transfection was demonstrated in both cell lines since the control lethal siRNA reduced viability by greater than 98% in all assays (**Figure S1**). However, silencing of *EAF2* did not sensitize HT-29 cells to either simvastatin or lovastatin (**Figure 1**). These results suggest that down-regulation of *EAF2* expression can modulate the cellular response to both cholesterol-lowering drugs in HCT-116 colon cancer cells.

**Figure 4:**
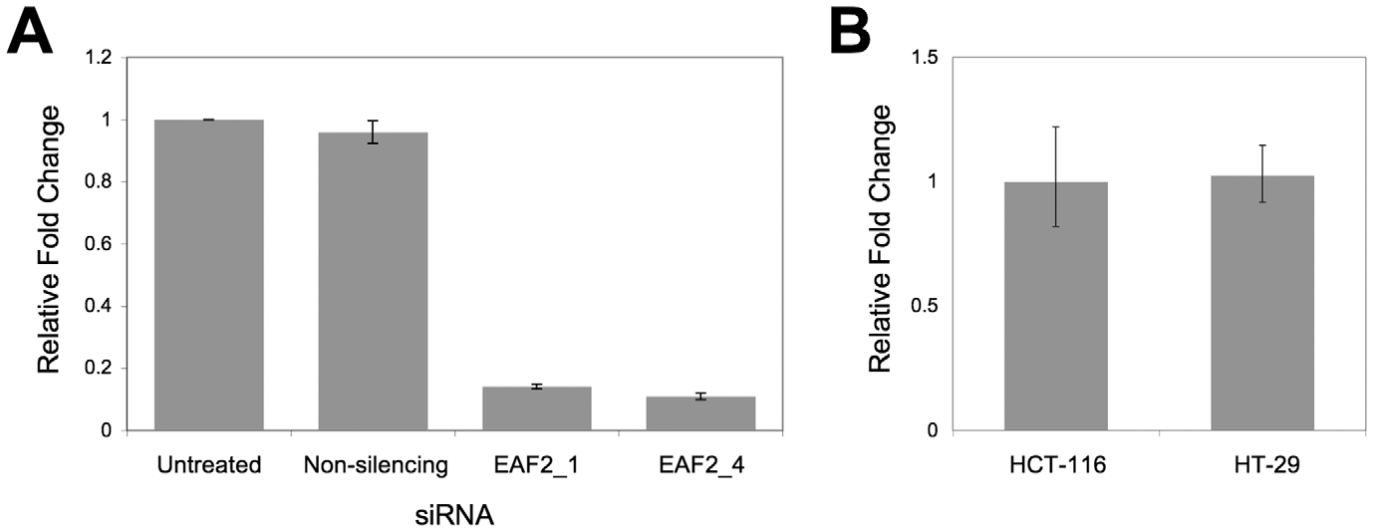
qPCR validation of gene silencing by EAF2 siRNA. (A) Total RNA was isolated from HCT-116 cells transfected for 48 hours with siRNA targeting *EAF2* (EAF2_1 and EAF2_4), non-silencing siRNA or untreated cells. Relative fold differences in *EAF2* mRNA levels compared to untreated and non-silencing siRNA treated cells are shown. (B) qPCR relative expression analysis of EAF2 in HCT-116 and HT-29 cells shows similar expression.

**Figure 5:**
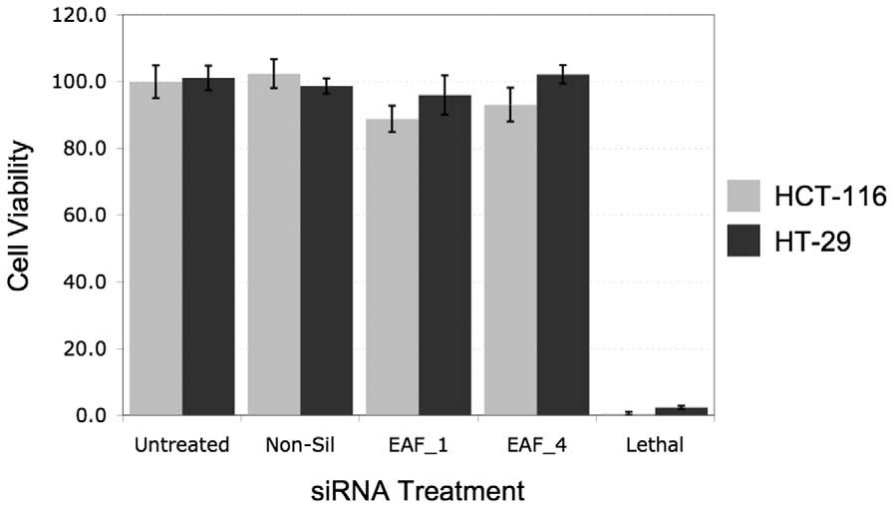
The effect of *EAF2* silencing on cell viability. HCT-116 cells and HT-29 cells (1000 cells/well) were reverse transfected with control siRNA and *EAF2* targeting siRNA. Cell viability was determined at 96 hours using Cell Titer Glo and read for luminescence. Data is represented as percent viability compared to non siRNA treated cells.

The efficacy of gene silencing by siRNAs EAF2_1 and EAF2_4 was confirmed using the qPCR experiments and is shown in **Figure 4A**. Even though HCT-116 cells showed sensitization to the combination of *EAF2* silencing and statin treatment, while HT-29 cells did not, both cell lines showed similar levels of expression of *EAF2* demonstrating that the difference in response was not due to difference in basal level of *EAF2* expression (**Figure 4B**). Moreover, siRNA silencing of *EAF2* in both cells lines had minimal effect on cell viability compared to untreated controls and the non-silencing siRNA control (**Figure 5**). Taken together, this data and the shift in the dose response curves produced by *EAF2* silencing in HCT-116 cells indicate that silencing *EAF2* potentiates the effect of statin induced cytotoxicity.

## Discussion

Our study represents the first systematic and whole-genome based association study for simvastatin and lovastatin, two statins that are promising candidates as cytotoxic drugs in cancer. In this study, we took advantage of the freely accessible and comprehensive pharmacological and genetic data on the NCI60 cell line panel to identify candidate genes that are associated with resistance to simvastatin and lovastatin in the human genome. Our results demonstrated the association of distinct sets of genes with resistance to these two drugs. We further functionally validated one target gene, *EAF2*, using siRNAs to show that its expression can modulate the cellular response to these two drugs in HCT-116 cell line.

A whole-genome SNP-based association study identified three genes associated with simvastatin resistance **(** **Table 3**). One of these genes, *NRP1* encodes for a membrane-bound receptor that has roles in angiogenesis, axon guidance, cell survival, migration, invasion and immune response. NRP1 also binds to SEMA3, whose activity is modulated by lipid rafts [28]. Another gene associated with simvastatin resistance was *COL13A1*, which encodes for the alpha chain of a nonfibrillar collagen located in the plasma membrane. Although its exact biological role has not been characterized yet, COL13A1 protein is located in adhesive structures of tissues and is thought to be involved in cell adhesion, migration and bone development [29]. Lastly, *MRPS31* encodes for a mitochondrial ribosomal protein that is involved in protein synthesis in mitochondria and is associated with type I diabetes [30].

In the case of lovastatin resistance, our analysis indicated the association of six genes (**Table 3**). One of these genes was *EAF2*, which is an androgen-response gene associated with the transcriptional elongation factor MEN/ELL and it is required for a variety of cellular functions such as the eye development and growth suppression and apoptosis induction [31], [32]. Recently, *Eaf2* knockout in a mouse model was associated with neoplasia of the lung, liver and prostate as well as B-cell lymphoma [33]. Interestingly, our results clearly show that silencing of *EAF2* decreases the GI_50_ values of HCT-116 colon cancer cells to not only lovastatin, but also simvastatin. This effect was not seen in the other colon cancer cell line HT-29 and this could be due to the genetic heterogeneity between the two cell lines. Another gene associated with lovastatin resistance was *ANK2,* which encodes for one of the three ankyrins that are involved in localization of proteins at the membrane. *ANK2* is critical for normal heart function and its mutations are one of the causes of congenital arrhythmia [34]. *AKAP7* encodes for a member of the A-kinase anchoring protein (AKAP) family that anchors the cAMP-dependent protein kinase A to specific subcellular compartments [35]. On the other hand, *LPIN2* is one of the three lipin genes. Lpin 1 is involved in adipose tissue development [36]. Although the exact biological role of this gene is not known, when mutated, this gene causes Majeed syndrome, an autoinflammatory disorder [37]. In addition, *STEAP2*, which encodes a multi-pass membrane protein that localizes to the Golgi complex, the plasma membrane, and the vesicular tubular structures in the cytosol, was also associated with resistance to lovastatin in our study. Recently a cupric reductase and ferrireductase role for STEAP2 protein was reported [38]. This gene is also implicated in prostate cancer [39]. Lastly, *PARVB*, a member of a focal adhesion protein family [40] that binds to the integrin-linked kinase [41] was also found associated with lovastatin resistance. It is interesting to note that the two lists of genes were vastly different between the two statins, which are both HMG-CoA reductase inhibitors. However, the two drugs are not identical and differ in their metabolites [42] and their cytotoxic effects [43], [44], which can explain these results. The cytotoxic effect mediated by the statins could involve targets other than HMG-CoA reductase such as the bone morphogenic protein (BMP) pathway as described by Kodach and colleagues [43].

We performed gene silencing and drug response experiments to test the validity of our GWAS results. In the case of three genes, *MRPS31*, *COL13A*, and *AKAP7,* our results did not confirm that their biological functions are directly related to resistance to simvastatin and lovastatin under our hypothesis and the experimental conditions applied. This discrepancy between the GWAS and functional studies can be explained by the possibility that these genes may represent false positive results of the GWAS analysis, or the conditions and the hypothesis applied were not optimum to detect the expected effects. Alternatively, these SNPs may be located in a genomic region that affects the function of distant genes, which cannot be tested using our approach. On the other hand, our genome wide association indicated the association of *EAF2* marker **1840275** (rs2332056, rs4339143) with resistance to only lovastatin after correction with FDR. In the case of simvastatin, this marker was associated with its resistance (unadjusted *p*<0.01), however, after correction for multiple testing, this significance was lost. Yet, our functional assessment using siRNA showed that silencing of *EAF2* was not only capable of modulating response to lovastatin but also to simvastatin in HCT-116 colon cancer cell line under the experimental conditions applied (**Figure 3**). This discrepancy between the results of genome wide association study and functional assessment experiments can be explained either by a false-negative association of *EAF2* marker in simvastatin set, or by utilization of different experimental conditions (e.g. drug dosage) in our siRNA experiments. Additionally, our results also showed that *EAF2* silencing did not sensitize another colon cancer cell line, HT-29 (which was considered relatively resistant to both simvastatin and lovastatin), suggesting the presence of a possible heterogeneity in genetic and molecular mechanisms involved in resistance to statins. Interestingly, HCT-116 is known to carry a *K-RAS* mutation while HT-29 does not [45]. Since the statins are inhibitors of HMG-CoA reductase that can lead to blocking farneslyation of K-RAS and thus K-RAS activation, it is possible that carrying a mutant *K-RAS* may contribute to making HCT-116 cells more susceptible to the combination of *EAF2* silencing and statin treatment. Further studies will be needed to address this possibility.

Our classification of both the HT-29 and HCT-116 being relatively resistant is based on the comparison to all the cell lines in the NCI60 panel. Previous studies on response to simvastatin and lovastatin of several of the NCI60 cell lines have been reported. The HL-60 leukemia cell line was previously found to be relatively resistant to simvastatin treatment (in a dose-dependent manner) than its all-trans retinoic acid resistant derivative cell line, HL-60-R2 [46]. Additionally, Martirosyan *et. al.* showed that the ovarian cancer cell line, SKOV-3, when treated with Lovastatin showed a response, but not as much as other cell lines included in their studies, suggesting that this cell line is not highly sensitive to lovastatin treatment under the conditions applied [47], which is in agreement with our results. Lastly, Kodach *et. al.* had also previously showed a dose-dependent cellular response to simvastatin and lovastatin in HT-29 and HCT-116 colon cancer cell lines [43]. Specifically, their findings suggest that at a low concentration, both statins increase the cell growth in HT-29, however, at higher concentrations, both statins reduce the cell growth rate in this cell line. In the case of HCT-116, the same group also found that this cell line was responsive to statin treatment at variable doses. Comparatively, they found that HT-29 was slightly more resistant to the statins than HCT-116, which is similar to our dose response data. For our GWAS study of the NCI60 data, these two cell lines were classified as relatively resistant to simvastatin and HT-29 was classified as relatively resistant to lovastatin. Therefore, these studies and our results should be interpreted cautiously, since the observed statin response may be modified by the experimental conditions and therefore may not be fully concordant among different experimental settings and among different studies. In addition, since resistance and sensitivity seems to be determined based on the comparison with other cell lines (which differs among the different studies), additional cautions should be exercised in interpretation of results.

Our data suggests that *EAF2* silencing can modulate the response of the statins in cancer cells such as HCT-116. The effect of *EAF2* silencing shifted the GI_50_ of the statins in HCT-116 cells by about 2–3 fold to low µM concentrations. These concentrations are higher than cholesterol controlling therapeutic plasma levels for lovastatin, which range from 50–200 nM [48]. However, higher doses of lovastatin, up to ten fold, have been tolerated for the treatment of cancer [49]. Further studies, such as in vivo studies, are needed to assess if *EAF2* silencing can modulate statin response at therapeutic doses.

### Conclusion

In conclusion, our results showed that two sets of distinct genes were associated with resistance to either simvastatin or lovastatin. Although they are both statins, resistance to them was determined by different genomic locations/genes. This finding suggests the different mechanisms and biology of resistance to these drugs. Moreover, we demonstrated that down-regulation in expression of *EAF2* could modulate the response to simvastatin and lovastatin in HCT-116 cells. Further studies are required to confirm the biological involvement of *EAF2* and other genes with simvastatin and lovastatin resistance and to determine the exact molecular basis of the drug resistance.

### Electronic Database Information

The URL addresses for the databases utilized in this study are as follows:

**dbSNP:** http://www.ncbi.nlm.nih.gov/SNP/

**DTP home page:** http://dtp.nci.nih.gov/index.html

**DTP Molecular Targets:** http://dtp.nci.nih.gov/mtargets/download.html

**Entrez Gene:** http://www.ncbi.nlm.nih.gov/sites/entrez?db=gene

**Gene Ontology:** http://www.geneontology.org/

**HapMap:** http://www.hapmap.org

**PLINK:** http://pngu.mgh.harvard.edu/~purcell/plink/index.shtml

**PUBMED:** http://www.ncbi.nlm.nih.gov/sites/entrez

## Supporting Information

Table S1 **List of NCI60 cell lines relatively resistant and relatively sensitive to simvastatin.** The standardized GI_50_ values from the NCI60 simvastatin data were analyzed by SAS 9.1. The visual antimode was used as a cut-off value at −0.2 for simvastatin to define sensitive (controls) and resistant (cases) NCI60 cell lines.
(PDF)

Table S2 **List of NCI60 cell lines relatively resistant and relatively sensitive to lovastatin.** The standardized GI_50_ values from the NCI60 simvastatin data were analyzed by SAS 9.1. The visual antimode was used as a cut-off value at −0.3 for lovastatin to define sensitive (controls) and resistant (cases) NCI60 cell lines.
(PDF)

Figure S1 **Effect of control siRNA treatment on the dose response to simvastatin and lovastatin.** HCT-116 cells (A & C) and HT-29 cells (B & D) were left untreated (Buffer) or transfected with control siRNA including Non-silencing sRNA and Lethal siRNA by reverse transfection. At 24 hours, the cells were treated with varying doses of either simvastatin (A & B) or lovastatin (C & D) ranging from 12 nM to 667 µM. Cell number was determined at 72 hours of drug exposure using Cell Titer Glo.
(TIF)

## References

[1] Lennernas H, Fager G (1997). Pharmacodynamics and pharmacokinetics of the HMG-CoA reductase inhibitors. Similarities and differences.. Clin Pharmacokinet.

[2] Kang S, Kim ES, Moon A (2009). Simvastatin and lovastatin inhibit breast cell invasion induced by H-Ras.. Oncol Rep.

[3] Gauthaman K, Fong CY, Bongso A (2009). Statins, stem cells, and cancer.. J Cell Biochem.

[4] Gauthaman K, Manasi N, Bongso A (2009). Statins inhibit the growth of variant human embryonic stem cells and cancer cells in vitro but not normal human embryonic stem cells.. Br J Pharmacol.

[5] Ogunwobi OO, Beales IL (2008). Statins inhibit proliferation and induce apoptosis in Barrett's esophageal adenocarcinoma cells.. Am J Gastroenterol.

[6] Fuchs D, Berges C, Opelz G, Daniel V, Naujokat C (2008). HMG-CoA reductase inhibitor simvastatin overcomes bortezomib-induced apoptosis resistance by disrupting a geranylgeranyl pyrophosphate-dependent survival pathway.. Biochem Biophys Res Commun.

[7] Freeman MR, Solomon KR (2004). Cholesterol and prostate cancer.. J Cell Biochem.

[8] Zhuang L, Lin J, Lu ML, Solomon KR, Freeman MR (2002). Cholesterol-rich lipid rafts mediate akt-regulated survival in prostate cancer cells.. Cancer Res.

[9] Hager MH, Solomon KR, Freeman MR (2006). The role of cholesterol in prostate cancer.. Curr Opin Clin Nutr Metab Care.

[10] Patra SK (2008). Dissecting lipid raft facilitated cell signaling pathways in cancer.. Biochim Biophys Acta.

[11] Abraham J, Earl HM, Pharoah PD, Caldas C (2006). Pharmacogenetics of cancer chemotherapy.. Biochim Biophys Acta.

[12] Cascorbi I (2006). Role of pharmacogenetics of ATP-binding cassette transporters in the pharmacokinetics of drugs.. Pharmacol Ther.

[13] Shoemaker RH (2006). The NCI60 human tumour cell line anticancer drug screen.. Nat Rev Cancer.

[14] Lorenzi PL, Reinhold WC, Rudelius M, Gunsior M, Shankavaram U (2006). Asparagine synthetase as a causal, predictive biomarker for L-asparaginase activity in ovarian cancer cells.. Mol Cancer Ther.

[15] Jarjanazi H, Kiefer J, Savas S, Briollais L, Tuzmen S (2008). Discovery of genetic profiles impacting response to chemotherapy: application to gemcitabine.. Hum Mutat.

[16] Savas S, Briollais L, Ibrahim-zada I, Jarjanazi H, Choi YH (2010). A whole-genome SNP association study of NCI60 cell line panel indicates a role of Ca2+ signaling in selenium resistance.. PLoS One.

[17] Eng L, Ibrahim-Zada I, Jarjanazi H, Savas S, Meschian M (2011). Bioinformatic analyses identifies novel protein-coding pharmacogenomic markers associated with paclitaxel sensitivity in NCI60 cancer cell lines.. BMC Med Genomics.

[18] Garraway LA, Widlund HR, Rubin MA, Getz G, Berger AJ (2005). Integrative genomic analyses identify MITF as a lineage survival oncogene amplified in malignant melanoma.. Nature.

[19] Purcell S, Neale B, Todd-Brown K, Thomas L, Ferreira MA (2007). PLINK: a tool set for whole-genome association and population-based linkage analyses.. Am J Hum Genet.

[20] Hochberg Y, Benjamini Y (1990). More powerful procedures for multiple significance testing.. Stat Med.

[21] Sherry ST, Ward MH, Kholodov M, Baker J, Phan L (2001). dbSNP: the NCBI database of genetic variation.. Nucleic Acids Res.

[22] Wheeler DL, Church DM, Lash AE, Leipe DD, Madden TL (2002). Database resources of the National Center for Biotechnology Information: 2002 update.. Nucleic Acids Res.

[23] Brattain MG, Fine WD, Khaled FM, Thompson J, Brattain DE (1981). Heterogeneity of malignant cells from a human colonic carcinoma.. Cancer Res.

[24] Fogh J, Trempe G, Fogh J (1975). New human tumor cell lines.. Human tumor cells in vitro.

[25] Azorsa DO, Gonzales IM, Basu GD, Choudhary A, Arora S (2009). Synthetic lethal RNAi screening identifies sensitizing targets for gemcitabine therapy in pancreatic cancer.. J Transl Med.

[26] Livak KJ, Schmittgen TD (2001). Analysis of relative gene expression data using real-time quantitative PCR and the 2(-Delta Delta C(T)) Method.. Methods.

[27] Tuzmen S, Kiefer J, Mousses S (2007). Validation of short interfering RNA knockdowns by quantitative real-time PCR.. Methods Mol Biol.

[28] Guirland C, Suzuki S, Kojima M, Lu B, Zheng JQ (2004). Lipid rafts mediate chemotropic guidance of nerve growth cones.. Neuron.

[29] Ylonen R, Kyronlahti T, Sund M, Ilves M, Lehenkari P (2005). Type XIII collagen strongly affects bone formation in transgenic mice.. J Bone Miner Res.

[30] Arden SD, Roep BO, Neophytou PI, Usac EF, Duinkerken G (1996). Imogen 38: a novel 38-kD islet mitochondrial autoantigen recognized by T cells from a newly diagnosed type 1 diabetic patient.. J Clin Invest.

[31] Maurus D, Heligon C, Burger-Schwarzler A, Brandli AW, Kuhl M (2005). Noncanonical Wnt-4 signaling and EAF2 are required for eye development in Xenopus laevis.. Embo J.

[32] Hahn J, Xiao W, Jiang F, Simone F, Thirman MJ (2007). Apoptosis induction and growth suppression by U19/Eaf2 is mediated through its ELL-binding domain.. Prostate.

[33] Xiao W, Zhang Q, Habermacher G, Yang X, Zhang AY (2008). U19/Eaf2 knockout causes lung adenocarcinoma, B-cell lymphoma, hepatocellular carcinoma and prostatic intraepithelial neoplasia.. Oncogene.

[34] Mohler PJ (2006). Ankyrins and human disease: what the electrophysiologist should know.. J Cardiovasc Electrophysiol.

[35] Gray PC, Scott JD, Catterall WA (1998). Regulation of ion channels by cAMP-dependent protein kinase and A-kinase anchoring proteins.. Curr Opin Neurobiol.

[36] Peterfy M, Phan J, Xu P, Reue K (2001). Lipodystrophy in the fld mouse results from mutation of a new gene encoding a nuclear protein, lipin.. Nat Genet.

[37] Al-Mosawi ZS, Al-Saad KK, Ijadi-Maghsoodi R, El-Shanti HI, Ferguson PJ (2007). A splice site mutation confirms the role of LPIN2 in Majeed syndrome.. Arthritis Rheum.

[38] Ohgami RS, Campagna DR, McDonald A, Fleming MD (2006). The Steap proteins are metalloreductases.. Blood.

[39] Porkka KP, Helenius MA, Visakorpi T (2002). Cloning and characterization of a novel six-transmembrane protein STEAP2, expressed in normal and malignant prostate.. Lab Invest.

[40] Korenbaum E, Olski TM, Noegel AA (2001). Genomic organization and expression profile of the parvin family of focal adhesion proteins in mice and humans.. Gene.

[41] Mongroo PS, Johnstone CN, Naruszewicz I, Leung-Hagesteijn C, Sung RK (2004). Beta-parvin inhibits integrin-linked kinase signaling and is downregulated in breast cancer.. Oncogene.

[42] Vree TB, Dammers E, Ulc I, Horkovics-Kovats S, Ryska M (2003). Differences between lovastatin and simvastatin hydrolysis in healthy male and female volunteers:gut hydrolysis of lovastatin is twice that of simvastatin.. ScientificWorldJournal.

[43] Kodach LL, Bleuming SA, Peppelenbosch MP, Hommes DW, van den Brink GR (2007). The effect of statins in colorectal cancer is mediated through the bone morphogenetic protein pathway.. Gastroenterology.

[44] Gbelcova H, Lenicek M, Zelenka J, Knejzlik Z, Dvorakova G (2008). Differences in antitumor effects of various statins on human pancreatic cancer.. Int J Cancer.

[45] Brink M, de Goeij AF, Weijenberg MP, Roemen GM, Lentjes MH (2003). K-ras oncogene mutations in sporadic colorectal cancer in The Netherlands Cohort Study.. Carcinogenesis.

[46] Tomiyama N, Matzno S, Kitada C, Nishiguchi E, Okamura N (2008). The possibility of simvastatin as a chemotherapeutic agent for all-trans retinoic acid-resistant promyelocytic leukemia.. Biol Pharm Bull.

[47] Martirosyan A, Clendening JW, Goard CA, Penn LZ (2010). Lovastatin induces apoptosis of ovarian cancer cells and synergizes with doxorubicin: potential therapeutic relevance.. BMC Cancer.

[48] Lewis KA, Holstein SA, Hohl RJ (2005). Lovastatin alters the isoprenoid biosynthetic pathway in acute myelogenous leukemia cells in vivo.. Leuk Res.

[49] Kim WS, Kim MM, Choi HJ, Yoon SS, Lee MH (2001). Phase II study of high-dose lovastatin in patients with advanced gastric adenocarcinoma.. Invest New Drugs.

