# Supplementary material for: NCI60 Cancer Cell Line Panel Data and RNAi Analysis Help Identify EAF2 as a Modulator of Simvastatin and Lovastatin Response in HCT-116 Cells
Source: PLoS One. 2011 Apr 4;6(4):e18306. doi: 10.1371/journal.pone.0018306 (PMC3070731; doi:10.1371/journal.pone.0018306)
Supplement: Table S1 — List of NCI60 cell lines relatively resistant and relatively sensitive to simvastatin. The standardized GI50 values from the NCI60 simvastatin data were analyzed by SAS 9.1. The visual antimode was used as a cut-off value at −0.2 for simvastatin to define sensitive (controls) and resistant (cases) NCI60 cell lines. (PDF) [file pone.0018306.s001.pdf]

Supplementary Table S1: List of sensitive and resistant NCI60 cell lines for Simvastatin.

Sensitive				Resistant			
CellPanelName	CellLineName	logValue	Normalized	CellPanelName	CellLineName	logValue	Normalized
NSCL	HOP-92	-8.523	-4.162785640	SCL	*DMS273	-5.540	-0.199477124
Renal	ACHN	-6.350	-1.275668843	Leukemia	HL-60(TB)	-5.522	-0.175561752
Melanoma	UACC-62	-6.329	-1.247767576	Colon	HCT-116	-5.485	-0.126402378
Melanoma	SK-MEL-2	-6.283	-1.186650516	Renal	UO-31	-5.429	-0.051999000
Renal	*RXF-631	-6.260	-1.156091985	Renal	CAKI-1	-5.395	-0.006825520
Ovarian	IGROV1	-6.189	-1.061759131	NSCL	A549/ATCC	-5.237	0.203098297
CNS	SNB-75	-6.182	-1.052458709	NSCL	*LXFL529	-5.176	0.284144834
CNS	U251	-6.116	-0.964769013	Colon	HT29	-5.164	0.300088415
Renal	*RXF393	-6.091	-0.931553219	Leukemia	RPMI-8226	-5.086	0.403721691
Renal	A498	-6.046	-0.871764790	Colon	KM12	-5.073	0.420993904
NSCL	EKVX	-6.023	-0.841206260	CNS	SF-539	-5.045	0.458195593
NSCL	NCI-H23	-6.008	-0.821276784	NSCL	NCI-H460	-5.042	0.462181488
Colon	HCT-15	-5.952	-0.746873406	NSCL	HOP-62	-5.036	0.470153279
CNS	SF-295	-5.947	-0.740230247	Renal	786-0	-5.030	0.478125069
Ovarian	OVCAR-8	-5.863	-0.628625180	Colon	SW-620	-5.018	0.494068650
Renal	SN12C	-5.821	-0.572822646	Leukemia	CCRF-CEM	-4.948	0.587072873
Melanoma	*M19-MEL	-5.807	-0.554221802	Colon	COLO205	-4.917	0.628260457
Ovarian	*SK-OV-3	-5.765	-0.498419268	Colon	*KM20L2	-4.911	0.636232248
Melanoma	M14	-5.648	-0.342969353	Leukemia	SR	-4.879	0.678748464
				Colon	HCC-2998	-4.783	0.806297112
				Melanoma	UACC-257	-4.763	0.832869747
				Leukemia	K-562	-4.755	0.843498801
				Ovarian	OVCAR-3	-4.636	1.001605980
				SCL	*DMS114	-4.626	1.014892297
				Leukemia	MOLT-4	-4.523	1.151741368
				NSCL	*HOP-18	-4.523	1.151741368
				NSCL	NCI-H322M	-4.523	1.151741368
				Colon	*DLD-1	-4.523	1.151741368
				CNS	*SNB-78	-4.523	1.151741368
				CNS	*XF498	-4.523	1.151741368
				Melanoma	SK-MEL-28	-4.523	1.151741368
				Ovarian	OVCAR-4	-4.523	1.151741368

NSCL: non-small cell lung carcinoma
CNS: central nervous system
SCL: small cell lung carcinoma
*These cell lines are not included in the GWAS analysis due to missing genotypes and/or other quality-control reasons.
