# Supplementary material for: NCI60 Cancer Cell Line Panel Data and RNAi Analysis Help Identify EAF2 as a Modulator of Simvastatin and Lovastatin Response in HCT-116 Cells
Source: PLoS One. 2011 Apr 4;6(4):e18306. doi: 10.1371/journal.pone.0018306 (PMC3070731; doi:10.1371/journal.pone.0018306)
Supplement: Table S2 — List of NCI60 cell lines relatively resistant and relatively sensitive to lovastatin. The standardized GI50 values from the NCI60 simvastatin data were analyzed by SAS 9.1. The visual antimode was used as a cut-off value at −0.3 for lovastatin to define sensitive (controls) and resistant (cases) NCI60 cell lines. (PDF) [file pone.0018306.s002.pdf]

Supplementary Table S2: List of NCI60 cell lines resistant and sensitive to Lovastatin.

Sensitive				Resistant			
CellPanelName	CellLineName	logValue	Normalized	CellPanelName	CellLineName	logValue	Normalized
NSCL	HOP-92	-7.511	-4.071633999	Colon	COLO205	-5.242	-0.234864309
CNS	U251	-6.260	-1.956253266	Ovarian	IGROV1	-5.225	-0.206118128
NSCL	NCI-H23	-5.993	-1.504769129	Renal	UO-31	-5.203	-0.168917188
CNS	SF-295	-5.936	-1.408384875	Melanoma	SK-MEL-5	-5.174	-0.119879585
Renal	CAKI-1	-5.933	-1.403312020	Colon	SW-620	-5.133	-0.050550560
Renal	A498	-5.919	-1.379638694	NSCL	*LXFL529	-5.110	-0.011658668
Melanoma	*UACC-62	-5.815	-1.203779704	Ovarian	OVCAR-8	-5.106	-0.004894861
Melanoma	LOXIMVI	-5.802	-1.181797331	SCL	DMS273	-5.061	0.071197972
Renal	SN12C	-5.767	-1.122614017	Leukemia	HL-60(TB)	-5.055	0.081343683
Renal	ACHN	-5.754	-1.100631643	CNS	*XF498	-5.034	0.116853671
Leukemia	SR	-5.675	-0.967046449	Renal	786-0	-5.010	0.157436515
Melanoma	*M19-MEL	-5.616	-0.867280291	Colon	HT29	-4.934	0.285948853
Renal	*RXF393	-5.599	-0.838534110	Ovarian	SK-OV-3	-4.928	0.296094564
Renal	*RXF-631	-5.594	-0.830079351	NSCL	NCI-H226	-4.926	0.299476468
CNS	SNB-75	-5.563	-0.777659844	CNS	SF-268	-4.912	0.323149793
Colon	HCT-15	-5.506	-0.681275590	Melanoma	M14	-4.910	0.326531697
				NSCL	HOP-62	-4.883	0.372187396
				Melanoma	SK-MEL-2	-4.878	0.380642155
				Colon	HCC-2998	-4.820	0.478717361
				Renal	TK-10	-4.790	0.529445916
				CNS	SF-539	-4.765	0.571719712
				NSCL	NCI-H460	-4.744	0.607229700
				Leukemia	CCRF-CEM	-4.736	0.620757315
				Leukemia	K-562	-4.709	0.666413014
				Melanoma	UACC-257	-4.700	0.681631580
				NSCL	A549ATCC	-4.673	0.727287280
				Melanoma	SK-MEL-28	-4.602	0.847344859
				Leukemia	MOLT-4	-4.572	0.898073414
				Leukemia	RPMI8226	-4.523	0.980930053
				NSCL	EKVX	-4.523	0.980930053
				NSCL	HOP-18	-4.523	0.980930053
				NSCL	NCI-H322M	-4.523	0.980930053
				SCL	*DMS114	-4.523	0.980930053
				Colon	*DLD-1	-4.523	0.980930053
				Colon	KM12	-4.523	0.980930053
				Colon	*KM20L2	-4.523	0.980930053
				CNS	*SNB-78	-4.523	0.980930053
				Melanoma	MALME-3M	-4.523	0.980930053
				Ovarian	OVCAR-3	-4.523	0.980930053
				Ovarian	OVCAR-4	-4.523	0.980930053
				Ovarian	OVCAR-5	-4.523	0.980930053

NSCL: non-small cell lung carcinoma
CNS: central nervous system
SCL: small cell lung carcinoma
*These cell lines are not included in the GWAS analysis due to missing genotypes and/or other quality-control reasons.
