# Supplementary figures and images for: NCI60 Cancer Cell Line Panel Data and RNAi Analysis Help Identify EAF2 as a Modulator of Simvastatin and Lovastatin Response in HCT-116 Cells
Source: PLoS One. 2011 Apr 4;6(4):e18306. doi: 10.1371/journal.pone.0018306 (PMC3070731; doi:10.1371/journal.pone.0018306)

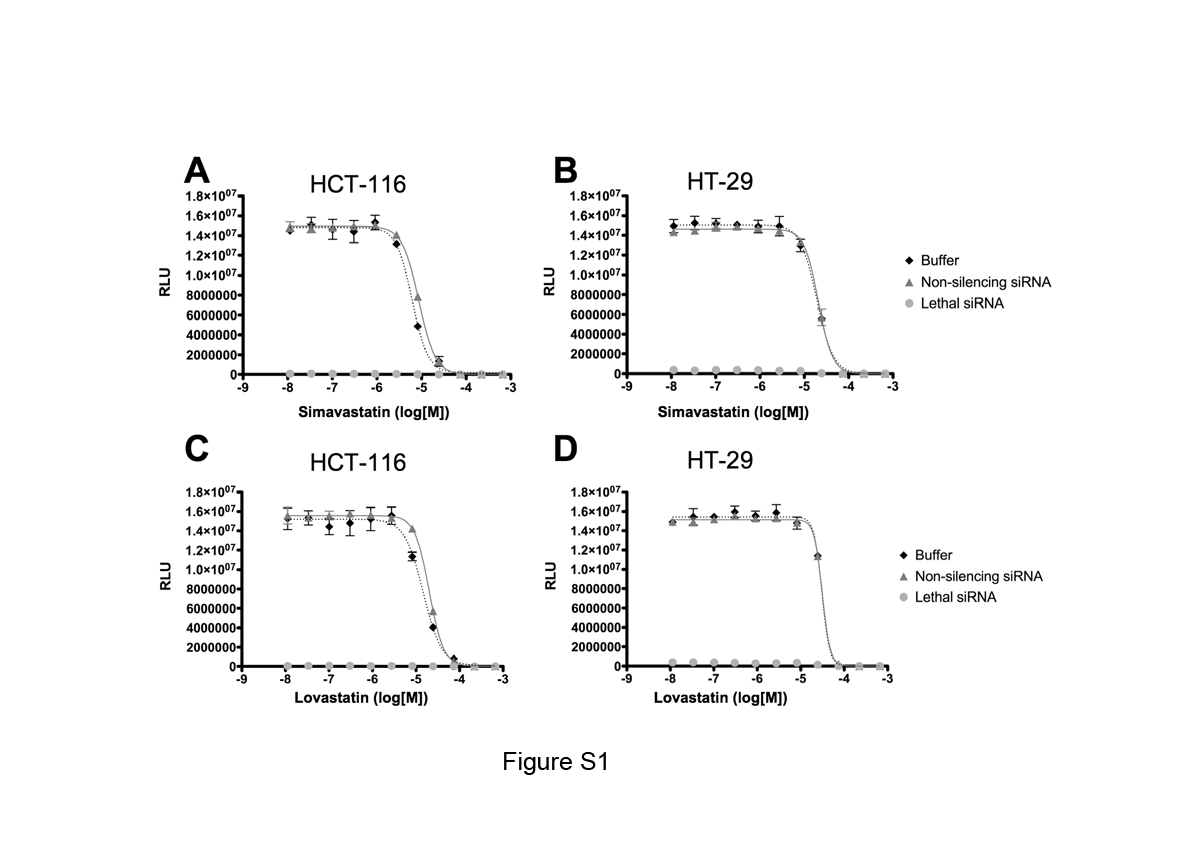

Supplement: Figure S1 — Effect of control siRNA treatment on the dose response to simvastatin and lovastatin. HCT-116 cells (A & C) and HT-29 cells (B & D) were left untreated (Buffer) or transfected with control siRNA including Non-silencing sRNA and Lethal siRNA by reverse transfection. At 24 hours, the cells were treated with varying doses of either simvastatin (A & B) or lovastatin (C & D) ranging from 12 nM to 667 µM. Cell number was determined at 72 hours of drug exposure using Cell Titer Glo. (TIF) [file pone.0018306.s003.tif]
